# Role of Kynurenine Pathway in Oxidative Stress during Neurodegenerative Disorders

**DOI:** 10.3390/cells10071603

**Published:** 2021-06-26

**Authors:** Adrian Mor, Anna Tankiewicz-Kwedlo, Anna Krupa, Dariusz Pawlak

**Affiliations:** 1 Department of Pharmacodynamics, Medical University of Bialystok, Mickiewicza 2c, 15-222 Bialystok, Poland; dariusz.pawlak@umb.edu.pl; 2 Department of Monitored Pharmacotherapy, Medical University of Bialystok, Mickiewicza 2c, 15-222 Bialystok, Poland; anna.tankiewicz-kwedlo@umb.edu.pl; 3 Department of Internal Medicine and Metabolic, Medical University of Bialystok, M. Skłodowskiej-Curie 24a, 15-276 Bialystok, Poland; anna.krupa@umb.edu.pl

**Keywords:** tryptophan, kynurenine pathway, kynurenine, 3-hydroxykynurenine, quinolinic acid, oxidative stress, reactive oxygen species, neurodegenerative disorders, excitotoxicity

## Abstract

Neurodegenerative disorders are chronic and life-threatening conditions negatively affecting the quality of patients’ lives. They often have a genetic background, but oxidative stress and mitochondrial damage seem to be at least partly responsible for their development. Recent reports indicate that the activation of the kynurenine pathway (KP), caused by an activation of proinflammatory factors accompanying neurodegenerative processes, leads to the accumulation of its neuroactive and pro-oxidative metabolites. This leads to an increase in the oxidative stress level, which increases mitochondrial damage, and disrupts the cellular energy metabolism. This significantly reduces viability and impairs the proper functioning of central nervous system cells and may aggravate symptoms of many psychiatric and neurodegenerative disorders. This suggests that the modulation of KP activity could be effective in alleviating these symptoms. Numerous reports indicate that tryptophan supplementation, inhibition of KP enzymes, and administration or analogs of KP metabolites show promising results in the management of neurodegenerative disorders in animal models. This review gathers and systematizes the knowledge concerning the role of metabolites and enzymes of the KP in the development of oxidative damage within brain cells during neurodegenerative disorders and potential strategies that could reduce the severity of this process.

## 1. Introduction

Neurological disorders are chronic and life-threatening conditions, negatively affecting the quality of human life. Their treatment options are still limited. In recent years, due to population growth and aging, their incidence has been constantly increasing, and therefore they have become a leading cause of disability and the second cause of death in the world [1,2,3]. The global prevalence of these disorders remains at a level of 10.2% [3]. The rate of disability and mortality associated with neurological disorders is significantly higher compared with other human disorders [3,4,5]. During pathological neurodegeneration, causative factors and cellular disturbances occur earlier, are much more intensified than in the aging-related processes, and often attack specific structures of the central nervous system (CNS). The abovementioned mitochondrial energy failure and excessive ROS production accompany the development of such diseases as Alzheimer’s disease (AD), Huntington’s disease (HD), and Parkinson’s disease (PD) [6,7,8,9,10,11,12,13].

Neurological disorders often have a genetic background, but in many cases, their progression is triggered or intensified by various exogenous factors [14,15,16]. Oxidative stress and mitochondrial damage seem to be at least partly responsible for their development [6,17]. The primary events responsible for the initiation of neurodegenerative disorders are still unclear, but the mechanism of oxidative damage seems to be significant in the propagation of cellular injuries observed at their beginning. Abnormal accumulation of protein deposits, such as amyloid-β protein, tau protein, α-synuclein, transactive response DNA-binding protein-43, and myelin debris, in and around neurons, is a histopathological hallmark of neurodegenerative processes. Their accumulation is unquestionably related to the pathogenesis and progression of neurodegenerative disorders. It induces molecular alterations, which in conjunction with activation of microglia and astrocytes, cause the release of pro-inflammatory cytokines, leading to the increased production of reactive oxygen (ROS) and nitrogen species, which damage the surrounding tissues and lead to neuroinflammation, most probably the last common pathway leading to neurodegeneration. Neuroinflammation potentiates the accumulation of the aforementioned abnormal proteinaceous materials in the brain tissue and enhances the neuroinflammatory conditions, forming a cyclic potentiation. [18]. Furthermore, mitochondrial DNA damage, enhanced by neuroinflammation and protein deposit accumulation, causes a loss of mitochondrial polypeptide expression, leading to a subsequent decrease in electron transport, an increase in ROS generation, disturbances of mitochondrial membrane potential, and finally to the release of cell death induction signals, which further amplify neuronal damage [7,19,20]. 

Loss of mitochondrial function also causes ionic imbalance, calcium (Ca^2+^) overload, and adenosine triphosphate (ATP) depletion. The potent drop of the energy supply induces the process of necrotic death. In turn, even mild or gradual energy disturbances may induce the release of proapoptotic factors from the mitochondria, leading to the initiation of an apoptotic cascade [8,21]. Moreover, excessive Ca^2+^ accumulation reveals a detrimental effect on cell viability, inducing oxidative damage of proteins and lipids and opening mitochondrial permeability transition pores, which also triggers the apoptotic cascade. However, it should not be forgotten that non-excitable cells, such as astrocytes and microglia, are strongly dependent on the intracellular Ca^2+^ concentration, and Ca^2+^ signaling is necessary to maintain their proper function [8,9,22,23]. Furthermore, the cellular processes observed during physiological aging are similar to those involved in the pathomechanism of neurodegenerative processes. 

The activation of the kynurenine pathway (KP) enzymes by interferon-γ, other proinflammatory cytokines, and cortisol links cell-mediated immunity, oxidative and nitrosative stress, and emotional stress with decreased serotonin levels and alterations in pain sensation and emotion regulation [11,12,13,24]. Some KP metabolites may enhance neuroinflammation and have detrimental effects on the mitochondria and energy metabolism. Therefore, they may generate oxidative stress, induce mitochondrial symptoms, and enhance neuronal dysfunction and apoptosis rate [11,12,13,24,25]. Measurement of redox status may reveal diagnostic, prognostic, or predictive value to diagnose and differentiate neurodegenerative disorders [26]. Therefore, the KP activity together with redox indicators seems to be a powerful battery of biomarkers in their diagnosis. Additionally targeting the components of the KP could be considered as a therapeutic solution, allowing efficient personalized treatment plans for this type of disorder to be built [27].

## 2. Role of Kynurenine Pathway Metabolites in Oxidative Stress-Associated Neurodegeneration

**Figure 1:**
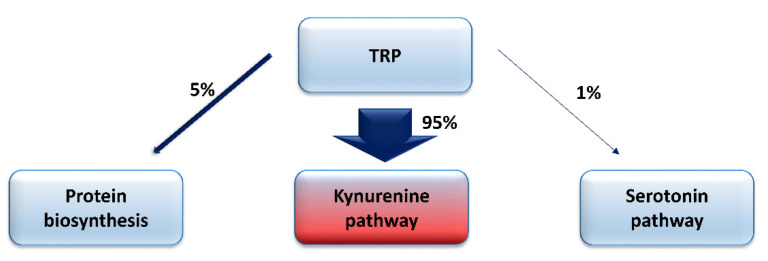
The main pathways of tryptophan (TRP) metabolism.

Tryptophan (TRP) is the essential amino acid involved in the process of protein biosynthesis. Considering its role in the development of neurodegenerative disorders, it cannot be forgotten that it undergoes extensive metabolism through several metabolic pathways, resulting in the production of many biologically-active compounds, exerting differential effects on numerous physiological and pathological processes [20,28,29,30,31,32,33,34,35,36,37,38,39,40,41,42,43,44,45,46]. TRP is transformed into serotonin in approximately 1% of its total body amount, while about 95% of this amino acid is metabolized by the KP, leading to the synthesis of the oxidized form of nicotinamide adenine dinucleotide (NAD+), a coenzyme participating in many cellular processes (Figure 1).

As mentioned above, the KP is the main path responsible for its catabolism. Its metabolites demonstrate diverse, sometimes opposite, activities in numerous biological processes [28,29,30,31]. Their accumulation may induce oxidative cell damage, which triggers the inflammatory process. They also modulate the activity of numerous signaling pathways, leading to the disruption of homeostasis and the functioning of various organs and systems. Thus, they contribute to the development of many systemic disorders, including neurological ones [11,32,33,34,35,36,37,38,39,40,41,42,43,44,45,46]. Within the KP, TRP is oxidized to N-formylkynurenine by tryptophan 2,3-dioxygenase (TDO) and two 2,3-indoleamine dioxygenase isoforms (IDO-1 and IDO-2) [47,48]. In physiological conditions, they catalyze the same reaction in parallel, but their tissue distribution is different [49,50,51,52,53]. N-formylkynurenine is catabolized into kynurenine (KYN), which is metabolized by the three branches of the KP, resulting in the formation of kynurenic acid (KYNA), anthranilic acid (AA), and 3-hydroxykynurenine (3-HKYN) [54,55,56,57]. The conversion of KYN to 3-HKYN occurs using kynurenine 3-monooxygenase (KMO), and to KYNA with the participation of kynurenine aminotransferase (KAT) [52,53]. 3-HKYN is metabolized into xanthurenic acid (XA) by KAT and then to 3-hydroxyanthranillic acid (3-HAA) by kynureninase (KYNU) [58,59,60]. The presence of enzymes that catalyze the transformation of both KYN to 3-HKYN and 3-HKYN to XA and 3-HAA was discovered in almost all tissues of the body [59,60,61]. 3-HAA is metabolized by 3-hydroxyanthranilic acid 3,4-dioxygenase (3-HAO) to aminocarboxymuconatesemialdehyde (ACMS), which is converted by ACMS decarboxylase to aminomuconicsemialdehyde. This compound undergoes non-enzymatic cyclization to picolinic acid (PA) or is non-enzymatically transformed to quinolinic acid (QA), which is converted by quinolinate phosphoribosyltransferase (QPRT) into NAD+ (Table 1) [61,62,63,64]. 

**Figure 2:**
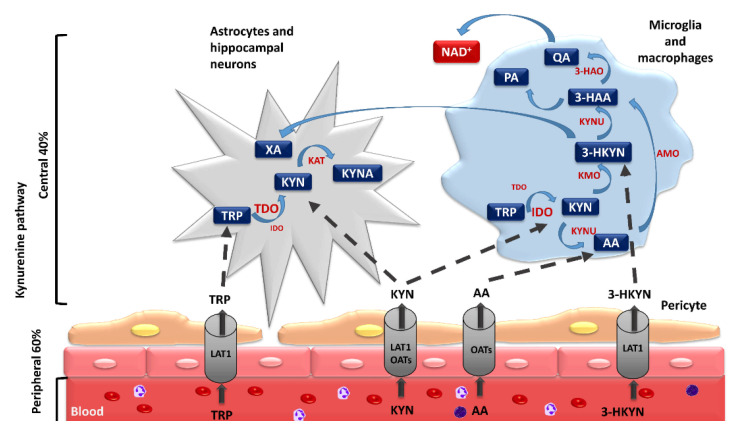
The course of the kynurenine pathway in various populations of the central nervous system cells. 3-HAA: 3-hydroxyanthranilic acid, 3-HAO: 3-hydroxyanthranilic acid 3,4-dioxygenase, 3-HKYN: 3-hydorxykynurenine, AA: anthranilic acid, AMO: aminocarboxymuconatesemialdehyde decarboxylase, IDO: indoleamine 2,3-dioxygenase, KAT: kynurenine aminotransferase, KMO: kynurenine 3-monooxygenase, KYN: kynurenine, KYNA: kynurenic acid, KYNU: kynureninase, LAT-1: large neutral amino acid transporter 1, NAD+: oxidized form of nicotinamide adenine dinucleotide, OATs: organic anion transporters, PA: picolinic acid, QA: quinolinic acid, TDO: tryptophan 2,3-dioxygenase, TRP: tryptophan, XA: xanthurenic acid.

Among the abovementioned metabolites, KYN, 3-HKYN, KYNA, AA, and QA turned out to be neuroactive [35,58,65,66]. KYN, 3-HKYN, and AA penetrate well through the blood–brain barrier (BBB), in opposition to KYNA, QA, and 3-HAA [24,67,68,69]. Further research indicated that 3-HKYN, 3-HAA, and QA have neurotoxic properties [24,35,65]. The peripheral increase in the activity of the KP, described by the elevated serum KYN/TRP ratio, was found in the course of numerous neurological and psychiatric disorders [24,70]. It is mainly associated with increased activity of peripheral IDO isoforms. Over 60% of the central KYN amount is delivered to the brain from peripheral circulation [71]. Peripheral KYN is transported through the BBB by the large neutral amino acid transporter 1 (LAT-1) as well as through organic anion transporters 1 and 3 (Figure 2) [72,73,74,75]. Therefore, it may easily enter into the CNS. In the brain, it is uptaken by glial cells, where it is metabolized. Neuroactive KP metabolites can directly or indirectly affect neuronal functions [72,73]. Abnormalities in their concentrations have been linked to numerous psychiatric and neurodegenerative symptoms [24,76,77]. However, it is still unclear if alterations of KP activity occur only during the progression of these disorders or also contribute to their initiation. Evaluation of the impact of the disruptions in KP activity on the pathogenesis of various neurological and psychiatric diseases will allow for a better understanding of their pathophysiology and the development of novel, effective therapeutic solutions. 

It is worth mentioning that KYN and KYNA are the main metabolites of the KP in the brain cells with a predominance of TDO activity, such as astrocytes and hippocampal neurons. In turn, in CNS cells with predominant IDO activity, such as microglia and macrophages, the authors observed further activation of the KP, leading to increased synthesis of 3-HKYN, recognized as a major inhibitor of neurogenesis (Figure 2) [72]. This method of KP activation depletes TRP, decreases serotonin and melatonin synthesis, and increases the production of active metabolites, which have deleterious effects on neuronal activity, survival, and the neurogenesis rate [78]. For this reason, numerous studies have focused on the possibility of KP activity modulation to reduce its detrimental impact on the CNS. The main strategies to restore and maintain a proper balance of the KP proposed by the authors include TRP supplementation, the use of inhibitors of KP enzymes, and the administration of analogs of KP metabolites, especially KYNA [61,79,80,81,82].

### 2.1. Tryptophan (TRP)

Several studies have already demonstrated that the administration of TRP may enhance oxidative stress levels and lead to an imbalance between the free radical content and the capacity of the cellular scavenging systems (Table 1) [83,84,85,86]. This effect is the most severe within the cerebral cortex. It can be induced both directly by an excess of TRP and by the accumulation of its active metabolites [83]. As mentioned above, changes in ROS levels may be associated with numerous alterations in brain cell functioning. Therefore, elevated TRP levels in the brain observed in the course of hypertryptophanemia and neurodegenerative disorders seem to be involved in the mechanisms leading to brain injury, observed in patients with these diseases. This detrimental effect could be diminished by antioxidant use. Although their use is a controversial issue, it seems to be reasonable that the administration of taurine or vitamins E and C might be useful as an adjuvant method in the treatment of patients with symptoms related to an excess of TRP [83,85,86]. Therefore, the impact of supplementation with vitamins E and C or taurine in these patients still needs further evaluation.

Despite the fact that excess TRP was linked with an increase in oxidative stress levels and severity of psychiatric and neurodegenerative symptoms, the first strategy was considered because, except for KYN, TRP is also metabolized to serotonin and melatonin [87,88,89,132]. Moreover, TRP supplementation has been widely used as an effective therapy to alleviate behavior problems in animals. A low-protein diet supplemented with TRP is a solution to manage excessive aggression in dogs [133]. The detailed mechanism associated with this effect of TRP is still unclear but seems to be at least partly associated with the impact of its neuroactive metabolites. In turn, Ciji et al. discovered that fish exposed to nitrites have increased cortisol serum levels and decreased testosterone and estradiol concentrations. This effect was prevented by the supplementation of vitamin E and TRP. The beneficial effect of vitamin E was associated with its antioxidant properties, but the exact mechanism responsible for the TRP action was also unknown. Its protective properties may result from both its direct redox properties as well as the impact of some of its antioxidant metabolites [134]. Additionally, TRP supplementation can also positively affect intracellular NAD+ concentrations, which improves cell viability and function, and protects cells against energy depletion [135]. However, a diet rich in TRP in healthy women increased the serum levels and urinary excretion of KYN, KYNA, 3-HKYN, 3-HAA, AA, and QA, the excesses of which are toxic. This indicates that redundant TRP supplementation could aggravate symptoms caused by oxidative stress due to the accumulation of its active metabolites [135,136].

### 2.2. Kynurenine (KYN)

KYN can scavenge hydrogen peroxide and superoxide in specific ROS producer systems. Increased KYN levels also lead to a decrease in ROS production by activated neutrophils [90]. Blanco-Ayala et al. showed that this compound, both in vitro and in vivo, reacts with hydroxyl radical and peroxynitrite, transforming into KYNA in these reactions [91,97]. In effect, it decreases the DNA and protein degradation process induced by these radicals. Additionally, KYN can attenuate the ROS production and lipid peroxidation induced by pro-oxidant compounds, such as iron(II) sulfate, peroxynitrite, and 3-nitropropionic acid, in rat brain homogenates [92]. Although KYN has been employed in many neurotoxic models as a neuroprotective agent, the observed effects were rather attributed to its metabolite, KYNA [11,92,93,97]. However, recent reports also suggest that they are at least partially induced by KYN’s own scavenging activity. This seems to be confirmed by the fact that KYN can donate electrons and protect macromolecules against oxidative modifications both in vitro and in vivo [94]. On the other hand, Song et al. claim that it cannot be ruled out that KYN, especially in excessive levels, may also contribute to apoptosis through ROS generation, because its co-incubation with antioxidants blocks cytochrome c release and activation of caspase 3, leading to an inhibition of apoptosis in a way associated with its activity (Table 1) [95]. 

### 2.3. Kynurenic Acid (KYNA)

Solvag et al. observed that plasma levels of KYNA are decreased in AD patients, suggesting the shift toward neurotoxic metabolites over neuroprotection in peripheral KP accompanying these diseases [137]. Interestingly, a similar trend was observed in PD and HD patients and children with autism spectrum disorders, while in vascular cognitive dementia, its serum concentration was increased [138]. This observation is extremely important because decreased levels of KYNA may provoke an inadequate anti-inflammatory response, resulting in enhanced tissue damage and exceeding cell proliferation during inflammatory in AD, PD, HD, and Lewy’s bodies dementia, contributing to the aggravation of their symptoms [98].

Brain levels of KYNA are elevated in brain tissue, mainly within the striatum and the hippocampus of AD patients [98,137,139]. In contrast, in HD patients, its levels are decreased in the precentral gyrus, frontal, and temporal cortex [98]. This compound acts mostly as a neuroprotectant and exerts a wide spectrum of endogenous antagonism on ionotropic excitatory amino acid receptors [12,13,97,140,141]. Its brain pool is mainly endogenous; however, a small part of brain KYNA is also derived from the peripheral circulation because it can cross the BBB through organic anion transporters 1 and 3 [142]. In micromolar concentrations, it acts as a competitive antagonist at the strychnine-insensitive glycine-binding site of the N-methyl-D-aspartate (NMDA) receptor and reveals a weak antagonistic effect on α-amino-3-hydroxy-5-methyl-4-isoxazolepropionic acid receptor (AMPAR) and kainite receptors in higher concentrations [97,141,143,144,145]. Furthermore, KYNA is capable of facilitating AMPAR responses in low concentrations, but the concentration range seems to be controversial. At the micromolar level, KYNA shows neuroinhibitory effects, while in nanomolar concentrations it turned out to be a facilitator of neurotransmission [97,141,145,146]. In addition to its direct impact on NMDA receptors, KYNA in a non-competitive way inhibits the 7-nicotinic acetylcholine receptors, in which presynaptic activation is involved in the regulation of NMDA release [97,141,147,148]. It has also been reported that KYNA, through the activation of the G protein-coupled receptor 35 (GPR35), can induce inositol trisphosphate synthesis and Ca^2+^ mobilization, but the significance of this phenomenon regarding the CNS is questionable, because GPR35 in the CNS has a low expression level, while relatively high KYNA concentrations are necessary for its activation [97,141,149]. Furthermore, KYNA significantly inhibited the in vivo transformation of hippocampal microglia cells from changing into the activated forms and the blood IL-6 level and significantly inhibited the in vitro phagocytosis of fluorescent microbeads in microglia cells induced by LPS treatment [150]. Moreover, KYNA also exhibits strong antioxidant activity by scavenging ROS in a way independent of NMDA and nicotinic receptor-associated mechanisms. In vitro models shows that in concentrations above 100 µM, KYNA can potently abolish ROS synthesis and prevent tissue damage triggered by overshooting inflammation (Table 1) [96,97,98]. However, an effect as potent as that observed at this concentration is difficult to observe in vivo, because its serum concentration observed in AD, HD, PD, and Lewy’s bodies dementia patients usually do not reach 70 µM, while in brain tissue homogenates it rarely exceeds the level of 10 µmol/g of protein [98,137,139].

### 2.4. 3-Hydroxykynurenine (3-HKYN) and 3-Hydroxyanthranillic Acid (3-HAA)

**Figure 3:**
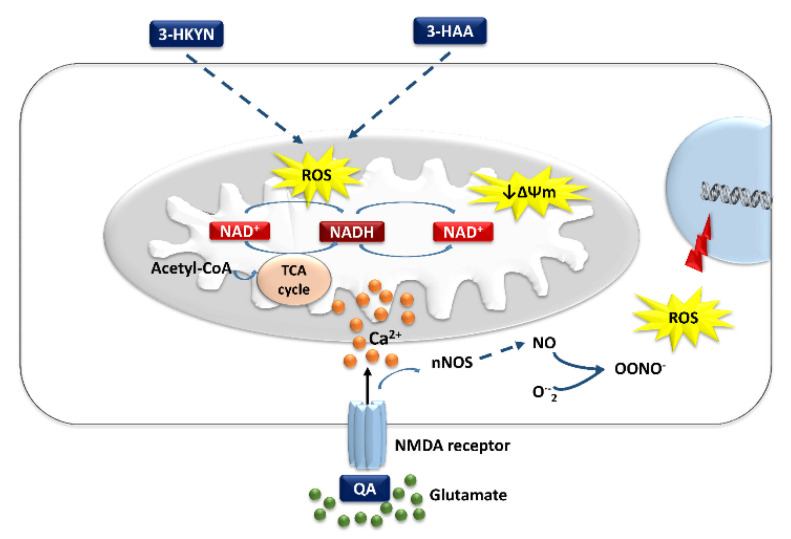
The involvement of 3-hydroxykynurenine, 3-hydroxyanthranilic acid, and quinolinic acid in the development of oxidative damages in the central nervous system cells. 3-HAA: 3-hydroxyanthranilic acid 3,4-dioxygenase, 3-HKYN: 3-hydroxykynurenine, acetyl-CoA: acetyl coenzyme A; Ca^2+^: calcium, NAD^+^: oxidized form of nicotinamide adenine dinucleotide, NADH: reduced form of nicotinamide adenine dinucleotide, NMDA: N-methyl-D-aspartate, nNOS: neuronal nitric oxide synthases, NO: nitric oxide, O_2_^•−^: superoxide, OONO^−^: peroxynitrite, QA: quinolinic acid, ROS: reactive oxygen species, TCA cycle: tricarboxylic acid cycle, Ψm: the mitochondrial membrane potential.

3-HKYN, an intermediate metabolite of KP, has been hypothesized to be a neurotoxic molecule, contributing to the development of various neurodegenerative lesions in the experimental models and clinical conditions [58,99,100,151]. The brain cortex and striatum are most sensitive to the impact of 3-HKYN, which is transferred into neurons through the LAT-1. Differences in the susceptibility to 3-HKYN in various brain regions result mainly from differences in LAT-1 expression in their cells [58]. Only by interacting with cellular xanthine oxidase can 3-HKYN generate sufficient amounts of ROS, such as superoxide radicals, hydrogen peroxide, and hydroxyl radical, to induce inter-nucleosomal DNA damage, leading to apoptosis [100,151]. However, even relatively low levels of 3-HKYN and 3-HAA may cause neurotoxicity via the induction of oxidative stress (Figure 3). They can promote the apoptotic death of neurons and enhance the severity of QA-induced excitotoxicity (Table 1) [58,101]. In addition, their excess is also associated with depressive symptoms [58,80,102]. The authors suggest a link between ROS overproduction and increased monoamine oxidase activity, which lowers brain levels of catecholamines and serotonin [152,153]. Moreover, polyunsaturated fatty acids are also sensitive to oxidation. ROS overproduction may damage phospholipids and reduce cell membrane viscosity [154]. Alterations in membrane viscosity may negatively impact the density and function of serotonergic or catecholaminergic receptors [152]. All these changes may intensify the development of depressive symptoms [155,156,157]. Various agents with antioxidant activity may prevent the detrimental effects induced by 3-HKYN and 3-HAA in neurons. Furthermore, selective serotonin reuptake inhibitors also revealed antioxidant properties and the ability to reverse the overproduction of ROS. This effect seems to enhance their antidepressant effect [158].

The neurotoxicity of KYN metabolites has been well demonstrated in vitro, in vivo, and in patients [11,13,25,33,35,55,56,58,65,103,107,108,139,151,158,159,160,161,162,163,164,165,166,167]. Preclinical and clinical data demonstrated that their elevated levels occur in several degenerative diseases. Increased synthesis of 3-HKYN and 3-HAA was confirmed in the course of AD, HD, and PD [11,12,13,24,33,56,58,151,159,160,161,162,163,164,165,166,167]. In the pathogenesis of AD, oxidative stress induced by their excess significantly enhances neuronal damage and may potentiate the neurodegenerative processes associated with the accumulation of Aβ-amyloid, glial activation, and upregulation of proinflammatory cytokine expression [139,159,160,161,162]. Additionally, the pathomechanism of HD may be at least partly associated with alterations in TRP metabolism and mitochondrial dysfunction. Significantly elevated concentrations of 3-HKYN and 3-HAA have been reported in the brains of HD patients, even at early stages of the disease [55,70,162,163,164,165,166,167]. Additionally, in the course of PD, decreased KYNA and increased 3-HKYN concentrations were found in brain samples [110,168,169,170,171,172,173,174]. Increased production of 3-HKYN and 3-HAA may contribute to the neuronal damage responsible for the cognitive disorders observed during aging, malaria, ischemia, traumatic injury, birth hypoxia, and epilepsy [11,56,175,176,177]. Furthermore, their accumulation may be involved in the development of various psychiatric diseases, such as anxiety, depression, and schizophrenia. All this evidence demonstrates that excess 3-HKYN and 3-HAA is at least partly responsible for the development of neurodegenerative and psychiatric symptoms [56,178,179,180].

It is worth noting that the issue of the impact of 3-HKYN and 3-HAA on the development of detrimental lesions in the brain cells associated with oxidative stress is controversial. The literature indicates the dual role of 3-HKYN in the CNS [99,104,105]. In vitro studies show that it has both neurotoxic and antioxidant properties, whereas in vivo studies suggest its role rather as a weak neurotoxin. However, there are some discrepancies in the research results. Colín-González et al. showed that 3-HKYN in rat striatal slices revealed a concentration- and time-dependent impact on lipid peroxidation, inducing both pro-oxidative at low (5–20 nM) and antioxidative properties at higher (100 nM) concentrations [99]. In turn, Braidy et al. achieved different results. They demonstrated that both in primary human astrocytes and neurons, 3-HKYN promoted NAD+ synthesis at concentrations below 100 nM, while at concentrations above 100 nM it caused a decrease in intracellular NAD+ levels and an increase in extracellular lactate dehydrogenase (LDH) activity, leading to a decrease in the viability of human cerebral neurons (Table 1) [104]. 

This phenomenon is important due to the fact that NAD+ biosynthesis is essential for the energetic balance and maintenance of cell viability and functions. However, it seems to be rather a form of a compensatory mechanism, because animals receiving 3-HKYN in striatal injection in high concentrations showed no behavioral or morphological changes in their striata. Moreover, in the range of high concentrations, 3-HKYN was unable to induce mitochondrial dysfunction in brain slices and prevented the detrimental lesions caused by its downstream metabolite, QA, the mitochondrial toxin 3-nitropropionic acid, and iron(II) sulfate. These protective properties were associated with the induction of glutathione S-transferase (GST) and superoxide dismutase (SOD) activities (Table 1) [99]. 

Moreover, 3-HKYN stimulates the synthesis and activation of Nrf2, a transcription factor and antioxidant regulator, and the proteins associated with this molecule, which caused a partial increase in cell resistance against oxidative stress [99,106]. Accordingly, 3-HKYN exhibited reductive properties at high concentrations, although the striatal tissue infused with 3-HKYN exhibited a significant increase in lipid and protein peroxidation levels in a short time after infusion. After long-time exposition to 3-HKYN, it substantially decreased. This seems to be associated with an increase in Nrf2 expression, as well as glutathione (GS) reductase and GST activity [99]. 

Altogether, despite the divergences observed by the authors, these findings suggest that although 3-HKYN may exert pro-oxidative effects under certain conditions, it is more likely to modulate redox activity and reduce the risk of cellular oxidative damage. This suggests that in physiological conditions, 3-HKYN appears to be rather a redox modulator than a neurotoxin. Additionally, 3-HAA revealed both and pro-oxidant effects. It can reduce ROS concentrations by the formation of complexes with iron, inhibiting its autoxidation and physiologically acting rather as redox modulator and antioxidant [11,109]. 

### 2.5. Anthranilic Acid (AA) and Xanthurenic Acid (XA)

AA is almost exclusively known for its antioxidant activity [11]. It revealed the ability to inhibit the citric acid cycle and respiratory chain complexes I–III from interfering with mitochondrial function. Moreover, similarly to 3-HAA, it downregulates ROS formation due to its iron-complexing properties [180]. In addition, it has anti-inflammatory and weak neuroprotective properties associated with its ability to form complexes with copper ions and to inactivate hydroxyl radicals [110,111,112]. In turn, xanthurenic acid, similarly to its precursor, 3-HKYN, revealed both pro-oxidative as well as antioxidative properties, based on similar mechanisms (Table 1) [113,114].

### 2.6. Picolinic Acid (PA)

PA was found to be a non-selective metal ion chelating agent and neuroprotectant. It revealed the ability to prevent QA-induced neuronal loss after injection of QA into the rat nucleus basalis magnocellularis and to activate brain macrophages. It inhibits excitotoxic but not neuroexcitatory responses within the CNS. Its mechanism of anti-excitotoxic activity is unclear but seems to be related to its zinc chelation capacity. Its effectiveness against QA-induced neurotoxicity was lower than KYNA but higher than AA. It also can modulate kainate-induced glutamate release from the striatum (Table 1) [115].

### 2.7. Quinolinic Acid (QA)

QA is a moderate, specific competitive agonist of the NMDA receptors. The hippocampus and striatum are the most sensitive to its neurotoxicity. The vulnerability to this compound observed in human cerebral neurons is associated with the fact that they can uptake exogenous QA in large amounts, but catabolize only its small part because QPRT is rapidly saturated [11,115,116]. There are two mechanisms responsible for the neurotoxic effect of QA on the glutamatergic system. The first one is associated with the direct overstimulation of the NMDA receptors by QA, which induces increased Ca^2+^ influx into the target neurons, leading to their damage, and death. It is named excitotoxicity, and neurons also affect oligodendrocytes. The second mechanism is associated with the ability of QA to contribute to ROS formation. The influx of Ca^2+^ into neurons induced by the activation of NMDA receptors contributes to the increase in the oxidative stress level (Figure 3) [93,116,117,119]. 

Furthermore, QA can induce oxidative damage in a manner independent of the NMDA receptor-associated mechanism. It creates complexes with iron(II) ions (QA–Fe^2+^). They enhance the formation of hydroxyl radicals. Especially lipid peroxidation induced by QA is intensified after an interaction with Fe^2+^ from these complexes. In addition, QA inhibits iron autoxidation due to their formation [117,120,121,122]. Both these pro-oxidative mechanisms lead to increased lipid and protein peroxidation levels, superoxide anion generation, and in effect disturbances in neuronal functioning and viability, especially within the hippocampus [116,117,118,119,120,121,122,123]. The direct pro-oxidative properties of QA expand its neurotoxic activity. Moreover, QA and 3-HKYN act synergistically in the production of ROS [124,125]. Therefore, even low doses of QA significantly potentiate the neurotoxicity of 3-HKYN. The QA–Fe^2+^ complexes also proved to be responsible for the breakage of DNA chains and the intensification of the lipid peroxidation process mediated by hydroxyl radicals observed in vitro (Table 1, Figure 3) [116,125]. 

In turn, Tronel et al. showed that hemin, a heme oxygenase 1 inducer, increases the neurotoxic effect of QA in the animal model and leads to an increase in the loss of brain tissue and microglia activation. This effect is mainly linked to increased ROS generation and iron accumulation [117,126]. Moreover, QA can enhance ROS synthesis by inducing nitric oxide synthases and intracellular poly(adenosine diphosphate (ADP)-ribose) polymerase activity in astrocytes and neurons, as well as activating extracellular LDH. This is confirmed by the fact that striatal slices exposed to QA showed a significant increase in both lipid peroxidation and LDH activity levels and a decrease in mitochondrial function (Table 1). These changes appear to be also related to the activation of cellular proteases [77,117,127]. 

Furthermore, in vivo studies proved that QA can modify the expression and activity of certain endogenous antioxidants in the brain, such as reduced GS and SOD. It can generate toxic peroxide radicals and peroxynitrite, even after a short-time impact on the cell [128]. It is worth mentioning that intracerebral injection of QA in the rat brain led to a significant increase in SOD expression levels in a time-dependent manner. This is recognized as a neuroprotective response to limit the oxidative damage caused by QA [129]. In support of this, it was found that QA infusion, in addition to increasing the ROS level, induces a delayed and prolonged upregulation in the antioxidant capacity in mice hippocampus. It also appears to be a cellular adaptive mechanism that may contribute to reducing oxidative stress levels [130]. Additionally, in synaptosomal fractions exposed to QA and 3-nitropropionic acid, even at nontoxic concentrations, Pérez-De La Cruz et al. observed a synergic increase in the oxidative stress levels, which was partially reduced by the administration of dizocilpine, a non-competitive antagonist of the NMDA receptor. This suggests that the severity of the lipid and protein peroxidation process in neurons induced by 3-nitropropionic acid, and probably by other pro-oxidants, is strongly dependent on cellular Ca^2+^ levels (Table 1). In turn, QA may increase the influx of Ca^2+^ into glutamatergic neurons [131]. Therefore, inhibitors of the NMDA receptors could be effective protectants against neurotoxic mechanisms induced or enhanced by QA.

The main symptom of AD is memory impairment. It is associated with the atrophy of the temporal lobes containing the hippocampal formations responsible for memory formation. The pathological processes within the hippocampus involve an excessive stimulation of excitatory glutamatergic neurons, especially the NMDA type. Although the NMDA receptors play an important role in the memory formation process, their overstimulation may cause neuronal dysfunction, damage, or even death. The accumulation of the Aβ-amyloid in AD brains causes several toxic effects, involving mitochondrial arrest, increased ROS generation, proteasomal dysfunction, as well as IDO induction [93,139,159,160,161,162,181,182]. QA seems to play an important role in the neuropathogenesis of AD due to its associations with inflammatory, pro-oxidative, and excitotoxic mechanisms. It can induce, among others, interleukin-1β synthesis and excessive NMDA transmission and inhibit GS function [159,181,182]. Additionally, in AIDS-associated dementia, the level of QA is increased in the cerebrospinal fluid and correlates with the severity of cognitive dysfunctions [183,184]. These findings can be useful for the development of novel therapies for AD and other neuroinflammatory diseases with accompanying dementia. In addition to for the upregulation of IDO isoforms and enhanced production of QA, the authors also confirmed the presence of elevated KYNA levels in the area of the putamen and caudate nuclei in AD patients. It appears to be a compensatory mechanism developed for counteracting the QA effects [182]. However, it may also contribute to the deterioration of cognitive functions due to the inhibition of the NMDA receptors. Interestingly, intrastriatal injection of QA in rodents causes neuropathological changes similar to those observed in the course of HD [185]. As mentioned earlier, QA can amplify the inflammatory response, mainly by the upregulation of several proinflammatory cytokine and chemokine receptor expressions. In vitro studies discovered that the potency of QA to induce inflammation is comparable to tumor necrosis factor-α or interferon-γ [181]. This indicates that its excess may worsen the course of numerous neuroinflammatory diseases.

In addition to the mechanisms described above, QA can exert early redox modifications also through alterations in the expression and activity of the Nrf2 transcription factor (Table 1). They are dependent on the time of exposure, the QA concentration, and the extent of oxidative damage occurring in the cells. Nrf2 can induce the upregulation of phase 2 enzymes, and therefore it seems to be a mechanism of compensatory response induced by QA-associated toxic processes in the cell. The loss of Nrf2 expression only moderately contributes to the severity of oxidative damage in cells, although striatal neurons from animals with this mutation showed higher sensitivity to the toxic effects of QA. In turn, the active role of the NMDA receptor in modulating oxidative stress and Nrf2-mediated response was observed only in the animals expressing this factor, but not in those with the loss of its function [186]. This indicates the need for further studies towards a better assessment of the impact of QA on Nrf2 activity and the potential role of this factor as a therapeutic tool for reducing neurodegenerative lesions associated with oxidative damage.

Numerous endo- and exogenous ROS scavengers, molecules with antioxidant properties, and activators of endogenous antioxidant enzymes revealed an efficiency against QA toxicity. The number of mechanisms and compounds that have evolved to counteract the adverse effects of this compound indicates the important role of the oxidative damage induced by QA in the development of neurodegenerative lesions [117]. Oxidative stress seems to be one of the most important mechanisms responsible for the QA-mediated neurotoxicity because free radicals can activate numerous signaling pathways, leading to extensive deleterious changes in the brain cells. Inflammation and oxidative stress are also involved in brain damage following a stroke. Increased KP activity contributes to an increase in oxidative stress levels by the impact of QA as well as other redox-active compounds, such as 3-HKYN and 3-HAA. The proinflammatory factors are detectable even in the early period following a stroke and proved to be associated with the rapid activation of the KP. Changes in the levels of 3-HKYN, 3-HAA, and QA appear to contribute to oxidative stress and brain damage after stroke. This indicates the need for the development of a new form of early anti-inflammatory interventions, effective in the modulation of the synthesis of such factors as KP metabolites, to reduce the inflammatory response, oxidative stress level, excitotoxicity, and in effect the development of delayed neurodegeneration [187]. Inhibitors of KP enzymes efficiently reduce ischemic damage in experimental models of stroke and brain inflammation in cerebral malaria [187,188,189,190]. Therefore, they could be useful for such interventions. In turn, the antioxidant properties of non-steroidal anti-inflammatory drugs, tolmetin and sulindac, can also protect hippocampal neurons against the oxidative damage induced by the accumulation of KP metabolites. Moreover, these agents prevent the depletion of reduced GS, decrease protein oxidation levels within the rat hippocampus, and improve spatial memory adversely affected by the accumulation of QA [191]. This finding supports the assumption that non-steroidal anti-inflammatory drugs may play a beneficial role in the management of neurodegenerative disorders.

## 3. Enzymes of the Kynurenine Pathway and the Possibility of Pharmacological Modulation of Their Activity

### 3.1. 2,3-Indoleamine Dioxygenase and 2,3-Tryptophan Dioxygenase

IDO-1 and IDO-2 participated in the oxidation of TRP to N-formylkynurenine, the first step of KYN synthesis. IDO-1 occurs in almost all body tissues. Its high activity was detected in the small intestine, spleen, lungs, epididymis, kidneys, blood vessel endothelium, and the brain. In physiological conditions, IDO-1 expression remains low, while interferon-γ, amyloid peptides, lipopolysaccharide, and other pro-inflammatory factors potently upregulate its expression and activation. IDO-2 plays a similar role and tissue distribution to IDO-1, but its physiological significance is still the subject of research [38,47,151,160,192]. 

The activation of IDO isoforms is mainly related to antioxidant capacities [11]. However, the downstream metabolites of KYN, which is a product of IDO, have both antioxidant and pro-oxidative properties. For example, as described above, 3-HKYN, 3-HAA, and QA play important roles in the development of various pathophysiological systemic disorders, including neurodegenerative ones, by the generation of oxidative and nitrosative damage, including lipid and protein peroxidation and excitotoxicity. Their excess enhances neuronal dysfunction and the apoptosis rate [193].

In the CNS, astrocytes play an important role in the maintenance of neuronal function and viability [192]. Inflammation within the CNS increases the concentration of pro-oxidative metabolites and the potential for NAD+ depletion through an increase in the poly (ADP-ribose) polymerase activity. On the other hand, the activity of IDO, the enzyme responsible for de novo NAD+ synthesis, is also significantly enhanced in astrocytes during inflammation [194,195]. Pretreatment of astroglial cells with interferon-γ increases IDO activity and intracellular NAD+ levels in vitro, leading to a decrease in their death ratio after exposure to hydrogen peroxide. These findings suggest that the induction of IDO and the subsequent de novo NAD+ synthesis may contribute to the maintenance of the proper intracellular energy supply and in effect cell viability and function in conditions of increased oxidative stress levels [194,196]. In turn, the inhibition of KP enzymes may in this case significantly decrease cellular functions and viability, unless alternate precursors for NAD+ synthesis are available (Table 2) [197]. This is confirmed by the fact that in vitro inhibition of IDO activity by pharmacological inhibitors, such as 1-methyl-L-tryptophan and QPRT using phthalic acid, resulted in a decreased intracellular NAD+ concentration and in effect cell viability both in astrocytes and neurons. Furthermore, this effect was more significant in neurons than astrocytes [11,198]. This suggests that changes in KP activity have a greater impact on the neurons than glial cells. It is also worth mentioning that IDO inhibition intensifies the progression of multiple sclerosis in a mouse model [196]. This seems to be associated with an increased CNS cell death rate caused by the decrease in NAD+ synthesis following IDO inhibition. 

However, some studies indicate that inhibition of KYN synthesis does not always induce deleterious effects. Jovanovic et al. indicated that an administration of 1-methyl-tryptophan seems to be a useful approach in migraine and neuropathic pain treatment. In turn, Kanai et al. demonstrated that TDO modulates anxiety-related behavior in mice, while Campesan et al. showed that inhibition of TDO activity, by silencing its gene, and protects against the severity of HD symptoms in the transgenic *Drosophila melanogaster* model [163,200,209]. In turn, Sorgdrager et al. proved that long-term administration of 680C91, the oral inhibitor of TDO, significantly reversed recognition memory deficits in the mouse model of AD, however without affecting spatial learning and memory or anxiety-related behavior [199]. Moreover, preclinical studies also found that its inhibition is effective in alleviating depressive symptoms, which are also, to some extent, associated with an increase in oxidative stress levels caused by inflammation and KP overactivity in neurons (Table 2) [68,151,200].

In turn, Bonda et al. showed that the levels of 3-HKYN and IDO activity were elevated in the brain samples taken from AD patients in comparison with healthy individuals. Importantly, the authors confirmed associations between IDO activation and senile plaque accumulation. Its activity was also related to the number of neurofibrillary tangles in neurons. Both factors are the pathological lesions typical for AD; therefore, the KP seems to be related to the development of destructive neurodegenerative changes observed in the course of this disease [160].

### 3.2. Kynurenine 3-Monooxygenase 

When cellular energy flow is disturbed, the activity of the KMO branch of the KP is enhanced [55,104]. This seems to be a compensatory mechanism, necessary to increase the NAD+ production required for ATP synthesis and proper functioning of the electron transport system in the mitochondria. While acute KMO activation may enable cells to temporarily meet higher energy demands, its chronic overactivity leads to oxidative damage and increases the apoptosis rate. Accordingly, prolonged KMO induction decreases the mitochondrial spare-respiratory capacity and increases ROS production. This indicates that KMO activity may have the opposite effects for neurons. Its moderate activation may promote favorable bioenergetics processes, but excessive activation would deplete energy stores and induce cell damage, leading to its dysfunction [104,201,202,203]. This mechanism seems to be important in terms of neurodegenerative diseases because neurons are highly sensitive to oxidative stress, energy depletion, and mitochondrial impairment (Table 2) [201,202,203]. Therefore, pharmacological inhibition of KMO activity seems to be a promising therapeutic tool in the management of various neurodegenerative disorders [104,163,201]. As mentioned above, the inhibition of IDO and QPRT activity may reduce the viability of astrocytes and neurons and exacerbate the clinical and histologic disease parameters during experimental autoimmune encephalomyelitis. In turn, preclinical studies indicate that targeting KMO in neurological disorders is a more efficient and safe solution [11,198]. 

In support of the above, Campesan et al. proved that KMO inhibition alleviates the severity of HD symptoms in the *Drosophila melanogaster* model (Table 2) [163]. Additionally, in the mouse model of HD, Sathyasaikumar et al. demonstrated a decrease in KYNU activity, the enzyme responsible for 3-HKYN degradation [162]. Treatment with the Ro 61-8048 and other KMO inhibitors, such as UPF648 and CHDI-340246, was effective in relieving neurological symptoms in the animal models of AD, HD, PD, and depression [55,162,163,209,210,211,212,213,214,215,216,217]. In turn, recent studies have shown that the administration of JM6, a new oral prodrug of Ro 61-8048, protects against behavioral deficits and synaptic loss, and, similarly to UPF648, increases the brain KYNA levels in the murine AD model as well as significantly inhibits microglial activation and prolongs life span in the mouse model of HD [218,219]. Therefore, KMO inhibitors seem to be a valuable therapeutic solution in the management of patients with AD, HD, and PD to ameliorate symptoms associated with 3-HKYN, 3-HAA, and QA accumulation in the brain. 

In addition, treatment with KMO inhibitors could be beneficial in other neurological disorders. It has been proven that Ro 61-8048 administration prevents ataxia and reduces the mortality of mice infected with the plasmodium parasite [189]. All these reports indicate the need for further research concerning the use of KMO inhibitors in the therapy of neurological disorders with accompanying accumulation of KP metabolites. Importantly, their clinical utility in these diseases is still limited because most of them do not cross the BBB [55,217]. Therefore, the development of novel compounds with this ability is crucial.

An increase in brain KYNA levels can be achieved by modulating KP enzyme activity. Nicotinylalanine, an inhibitor of KYNU and KMO, administered together with KYN and probenecid, which is an inhibitor of organic anion transporters 1 and 3, significantly increases brain KYNA levels and reduces the neurotoxic symptoms induced by QA [172,204,220,221,222,223]. Giorgini et al. found that the administration of Ro 61-8048, a high-affinity KMO inhibitor, significantly reduced 3-HKYN production in vitro. However, its effectiveness seems to be limited because it did not inhibit QA synthesis nor alleviate ROS generation [212]. What is interesting, Amori et al. showed that the selective inhibition of KMO in vivo, using UPF648, leads to a decrease in the brain levels of both 3-HKYN and QA and a moderate increase in KYNA synthesis. This effect was observed even in animals pretreated with intrastriatal QA injection [55]. Moreover, in vitro research also proved that a loss of function mutation of KMO significantly reduced the toxicity of the mutant huntingtin fragment in a yeast HD transgenic model due to silencing the mechanism associated with ROS generation [212]. All of this indicates that the inhibition of 3-HKYN synthesis allows the severity of pathological changes during the neurodegenerative process to be reduced; therefore, the targeting of KMO could be an effective way to treat such diseases.

### 3.3. Kynurenine Aminotransferase and 3-Hydroxyanthranilic Acid 3,4-Dioxygenase

Although KAT isoform activity and brain KYNA levels were decreased in patients with neurodegenerative disorders, and this compound is widely recognized as a neuroprotectant [91,203,224,225], preclinical studies have demonstrated that excessive levels of both neurotoxic QA as well as neuroinhibitory KYNA can have detrimental effects on CNS cell function. KAT II inhibition by BFF816, although it decreases brain KYNA levels, may enhance cognitive abilities (Table 2) [73,204,205,206,207]. Yates et al. observed that the administration of 4-chloro-3-hydroxyanthranilate, a synthetic inhibitor of 3-HAO, leads to a decrease in QA synthesis and alleviates functional deficits in the experimental model of spinal cord injury in guinea pigs (Table 2) [208]. In turn, Vallerini et al. discovered that 2-aminonicotinic acid 1-oxide derivatives are stable, low molecular weight, and BBB-permeable inhibitors of 3-HAO. The most active of them can effectively shift the flux of the KP from QA toward KYNA in neurodegenerative conditions [226]. The downstream enzymes of KP appear to be a novel tool for better understanding the impact of the KP in the development of neurodegenerative disorders, such as AD, HD, epilepsy, and amyotrophic lateral sclerosis. Recent in vivo studies suggest that targeting them may open up new treatment options for the aforementioned diseases.

Excess 3-HKYN and its metabolites, 3-HAA and QA, can impair ATP production, and reduce the respiratory control index and the ADP/oxygen ratio in mitochondria. This fact justifies KP enzyme inhibitor use to counteract these effects [163,227]. KMO inhibition appears to be the most promising solution in preventing the accumulation of 3-HKYN, 3-HAA, and QA, and consequently interleukin-1β-dependent reduction of neurogenesis. In turn, the impact of the proinflammatory cytokines on KP metabolite concentration and their relationship with the neurogenesis rate indicates an important role of the KP in an inflammation-induced decrease in neurogenesis [70,224,228,229,230,231].

## 4. Analogs of Kynurenine Pathway Metabolites

Over the past years, several types of analogs of KP metabolites have been developed. Most of them belong to synthetic derivatives of KYNA. Their halogenated analogs, such as 7-Cl-KYNA, showed potent neuroprotective properties. However, their therapeutic significance is still limited, because most of them have poor BBB permeability. To date, only a few compounds, such as SZR72, SZR81, SZR104, and a prodrug 4-Cl-KYN, revealed the ability to cross this barrier. Therefore, this compound has the greatest potential to be used in the management of neurodegenerative disorders such as AD [13,232,233].

Synthetic analogs of KYNA are an important and numerous part of the several types of analogs of KP metabolites that have been developed over the last years [13,232]. A novel one, a KYNA amide derivative, 2-(2-N,N-dimethylaminoethylamine-1-carbonyl)-1H-quinolin-4-one hydrochloride, exhibits similar neuroprotective and neuroinhibitory properties to KYNA [13,81]. It showed beneficial effects in numerous areas. In the micromolar range, it significantly lowers the amplitude of the excitatory postsynaptic potentials within the hippocampus CA1 region. In turn, at the nanomolar level, it exerts a facilitatory effect on neurotransmission [81]. Similar to KYNA, it is capable of facilitating AMPAR responses. In the transgenic mouse model of HD, its administration prolongs survival time and prevents loss of striatal neurons [234]. Assessment of its anti-inflammatory properties proved that it has a greater ability to inhibit tumor necrosis factor-α synthesis than endogenous KYNA, which suggests its potent immunoregulatory properties [235]. It also effectively reduces oxidative stress levels associated with inflammation development and revealed a neuroprotective effect against ischemia-induced neuronal loss. Although its exact mechanism is unknown, the lack of cognitive adverse effects makes this compound a promising candidate for further investigations [236,237]. It could be useful in the management of neurodegenerative disorders such as AD and HD.

Halogenated analogs of KYNA, such as 7-Cl-KYNA, demonstrated high effectiveness as neuroprotectants because they are a selective antagonist of the glycine site of NMDA receptors and significantly reduce kainite-induced seizure activity [82,233,238]. Another synthetic KYNA analog, 5,7-ichlorokynurenic, despite being a potent antagonist of NMDA, does not decrease the level of hyperphosphorylation induced by protein phosphatase 2 inhibition, detrimental to neuron viability [239]. The clinical significance of most of them is questionable because their bioavailability within the CNS is poor [232]. Therefore, researchers focused on the development of agents with this property. It led them to the discovery of 4-Cl-KYN, a prodrug that enters the brain cells, where it is transformed into 7-Cl-KYNA by endogenous KAT isoforms. Systemic administration of 4-Cl-KYN leads to a significant increase in 7-Cl-KYNA levels in hippocampal neurons. Finally, it effectively alleviates the neurotoxic effects of QA in the rat hippocampus and striatum. This compound has completed a phase I clinical trial. It passed the evaluation of safety, tolerability, and pharmacokinetic profile. Therefore, it has a good chance to enter clinical use in the treatment of neurodegenerative diseases [81].

The development of KYNA analogs exerting complex anti-excitotoxic properties and capable of crossing the BBB was the main goal of the research on compounds belonging to the SZR series [240]. SZR72, SZR81, and SZR104 seem to be the most promising agents from this group. They demonstrated the BBB permeability and significantly inhibited tumor necrosis factor-α synthesis in vitro [240,241,242]. Furthermore, administration of SZR72 downregulated protein kinase-like endoplasmic reticulum kinase and IL-1β activation in the trigeminal ganglions in Sprague–Dawley rats, while SZR81 reduced immobility time and increased swimming time in Charles Dawley mice [150,241,242,243]. It suggests that these compounds have potential in the treatment of migraines and depression [241,242]. In turn, SZR104, the newest representative of this family, significantly decreased the number of microglial cells in the epileptic hippocampus and prevented microglia proliferation and decreased microglial phagocytosis in vitro. However, it did not prevent behavioral convulsions and did not decrease the mortality in the epilepsy mice model. This suggests that it may not cross the BBB in vivo [240]. 

## 5. Aryl Hydrocarbon Receptor and Oxidative Stress

The toxic effects of polycyclic aromatic hydrocarbons, dioxins, and other compounds with a similar chemical structure are mainly mediated by the activation of an aryl hydrocarbon receptor (AhR). Research indicates that it operates beyond xenobiotic metabolism and exerts numerous pleiotropic functions. Physiologically it plays an important role in the regulation of the numerous cellular signaling pathways and is responsible for the maintenance of immune homeostasis. Its activation upregulates the IL-6 and signal transducer and activator of transcription 3 expressions, and in effect it induces the general control nonderepressible-2 kinase and mammalian target of rapamycin kinase pathways. In this manner, increased synthesis of KYN, mainly by IDO, suppresses the immune system response. It causes the inactivation and apoptosis of TH1 and effector T cells, as well as activation of immunosuppressive T regulatory cells, preventing an excessive immune response [43,44,46,244,245]. The excessive activation of AhR, caused by elevated concentrations of KYN and its metabolites, increases oxidative stress levels, proinflammatory cytokine release, and as a result enhances cell aging and death rate [32,42,43,46,245]. In the transgenic AhR-null mouse model, Garcia-Lara et al. observed elevated KYNA levels in specific brain areas. This suggests that AhR activity inhibition leads to an increase in the brain amount of neuroprotective KYNA and contributes to its effectiveness against excitotoxicity and oxidative stress within the CNS. Furthermore, AhR is a physiological receptor for KYN and KYNA, while KYNA has a higher affinity to AhR than KYN. Thus far, these compounds have been considered neuroprotectants and antioxidants. Furthermore, AhR also links them to a cascade of toxic processes leading to brain damage, observed in, among others, HD [245]. These data suggest that the pharmacological inhibition of AhR activity could be another interesting option for reducing the deleterious effects of excess KYN and its metabolites on neurons and has potential in the treatment of neurological disorders, including HD.

## 6. Summary and Therapeutic Perspective

Oxidative damage causes harmful alterations in the functioning of neurons. It can be prevented through antioxidant use [6,7,8,9,10,11,12,13]. In the course of hypertryptophanemia or neurodegenerative disorders, the oxidative stress associated with excess TRP and its metabolites may contribute to the mechanisms leading to brain injury. Although the use of antioxidant supplementation is a controversial issue, it appears to be reasonable that the administration of taurine or vitamin C and E supplementation in patients with symptoms associated with TRP accumulation could be useful as an adjuvant procedure during their therapy, due to their antioxidant properties [83,84,85,86]. It is worth mentioning that despite evidence of the pro-oxidative effects of this amino acid, Ciji et al. demonstrated that its administration could reduce the oxidative stress induced by exposition to nitrites. However, its exact mechanism in this case is unclear. Its protective properties might be the result of its own redox properties as well as the impact of its antioxidant metabolites [134]. 

Various compounds with antioxidant capacity prevent cell death induced by 3-HKYN and its metabolites [117,187]. The antioxidant properties of non-steroidal anti-inflammatory drugs, tolmetin and sulindac, can protect hippocampal neurons against 3-HKYN-, 3-HAA-, and QA-induced oxidative damage. This finding supports the hypothesis that the use of non-steroidal anti-inflammatory drugs could be effective in the treatment of various neurodegenerative disorders, such as AD and HD [191]. In turn, the inhibition of IDO and QPRT activities by 1-methyl-tryptophan and phthalic acid, respectively, leads to a decrease in intracellular NAD+ levels and cell viability, both in astrocytes and neurons. Therefore, their use in the treatment of neurodegenerative diseases is questionable [11,196,198]. However, it is worth mentioning that inhibition of IDO activity has some potential in neurological disorder therapy. Recent reports indicate that 1-methyl-tryptophan appears to be a promising agent for migraine and neuropathic pain therapies [209,246].

Inhibition of KMO by Ro 61-8048 significantly reduces 3-HKYN synthesis induced by the mutant huntingtin in the transgenic yeast model [212]. Moreover, its administration leads to an increase in brain KYNA levels in the PD monkey model [211], while in combination with levodopa, it reduces the severity of dyskinesias, both acute and after long-time administration. In turn, the combination of the KMO inhibitor with KYNA supplementation reveals a neuroprotective effect in the mouse model of PD [247]. Much preclinical research has proved that the administration of Ro 61-8048, UPF648, and CHDI-340246 was effective in the management of neurodegenerative and psychiatric disorders such as AD, HD, PD, and depression [55,162,163,209,210,211,212,213,214,215,216,217]. In turn, the oral prodrug JM6 ameliorated anxiety-related behavior, memory deficits, and synaptic loss, and increased the brain KYNA levels in the murine model of AD. It also efficiently decreased microglial activation and extended life span in transgenic mice with HD [218,219]. In addition to decreasing 3-HKYN and QA, treatment with UPF648 moderately elevated KYNA synthesis in rat brains [248]. In turn, in the mouse model of malaria, Ro 61–8048 averted ataxia and decreased the mortality rate [178]. The clinical utility of most of the currently available KMO inhibitors in brain disorders is limited because their BBB permeability is very weak [217,249]. Meanwhile, the novel competitive KMO inhibitors belonging to derivatives of aryl pyrimidine carboxylic acid can penetrate the BBB. Thus, they could be effective in the treatment of neurodegenerative diseases accompanied by alterations in KMO activity [55,217,249].

What is interesting is that the activation of KAT II in neurons appears to have an impact on such processes as programmed cell death. N-acetylcysteine, a brain-penetrant drug with antioxidant properties and procognitive effects in humans, increases the brain levels of reduced GS and indirectly inhibits KAT II. This mechanism seems to enhance the beneficial neuropharmacological effects of this drug and could be potentially useful in alleviating cognitive deficits in schizophrenia and other major brain diseases, such as AD [250]. 

Inhibition of 3-HAO activity by 4-chloro-3-hydroxyanthranilate and 2-aminonicotinic acid 1-oxide derivatives leads to a significant decrease in QA synthesis and alleviates the deleterious effects associated with its accumulation. This indicates that the inhibitors of downstream KP enzymes could be an effective pharmacological tool in the treatment of such neurodegenerative diseases as AD, HD, epilepsy, and amyotrophic lateral sclerosis [226,227]. Moreover, pharmacological inhibition of AhR could be a valuable option for a reduction of the harmful impact of excess KP metabolites on brain cells and has potential in the treatment of HD [245]. In turn, combined administration of KYN and probenecid increased the cortical levels of KYNA. This combination attenuated morphological lesions and improved spatial memory in the mouse model of AD. These compounds can also be co-administered with nicotinylalanine, an inhibitor of KYNU and KMO. In this setting, they relieve QA-induced changes in animal behavior [172,220,221,222,223]. Moreover, mitigation of the toxic effects caused by excess QA in the brain could be achieved using synthetic analogs of KP metabolites, such as 4-Cl-KYN, a BBB-permeable prodrug of 7Cl-KYNA [81,233].

## 7. Conclusions

Much research has proved the accumulation of neuroactive metabolites of the KP within the brain during numerous neurodegenerative and psychiatric disorders. Some of them are neurotoxic and stimulate the development of detrimental processes within the CNS. They can induce excitotoxicity, oxidative damage, and inflammation, leading to neuronal dysfunction and death. Preclinical studies indicate that strategies including targeting KP activity using TRP supplementation, KP enzyme inhibitors, or analogs of KP metabolites appear to be noteworthy therapeutic options against the excitotoxicity and oxidative stress accompanying neurological disorders as well as a neuroprotective solution. The greatest number of preclinical reports indicate that the use of KP enzyme inhibitors brings the most promising results in the treatment of these diseases. However, there are still many unknowns, and clinical solutions are far from being developed. The role of KP metabolites, oxidative stress, and excitotoxicity in the development of CNS pathologies still require better understanding to develop novel therapies effective in the management of neurodegenerative disorders. This indicates the need for further research on this topic, especially in terms of clinical application.

## Figures and Tables

**Table 1:** Impact of kynurenine pathway metabolites on oxidative stress during the development of neurodegenerative processes.

Metabolite	Role		References
	In Vitro	In Vivo	
**Tryptophan (TRP)**	- Possesses redox properties - Reacts readily with hydroxyl radical and singlet oxygen to form multiple oxidation products - Induces intracellular oxidized form of NAD (NAD^+^) synthesis	- Induces oxidative stress, an imbalance between free radical content and the capacity of the scavenging systems, in the cerebral cortex of the rats	[83,84,85,86,87,88,89]
	Despite evidence of the pro-oxidative effects, its administration could reduce the oxidative stress level. However, its exact mechanism is unclear. The observed result could be also induced by its metabolites’ impacts.		
**Kynurenine (KYN)**	- The neuroprotective effect presented by KYN is at least partially dependent on its scavenging activity - Decreases ROS production lipid peroxidation in rat brain homogenates below basal levels - Reacts with hydroxyl radical and peroxynitrite, generating kynurenic acid in these reactions - Can scavenge hydrogen peroxide and superoxide, and in effect reduce oxidative damage - Its excess induces apoptosis through ROS generation, since its co-incubation with an antioxidant blocks cytochrome c release and caspase 3 activation downregulating this apoptosis pathway	- Reacts with hydroxyl radical and peroxynitrite, generating kynurenic acid in these reactions	[11,12,13,90,91,92,93,94,95]
	Showed neuroprotective, and antioxidative properties, the observed effects were rather attributed to its metabolite, KYNA. Its excess may induce detrimental effects, enhancing ROS-dependent apoptosis.		
**Kynurenic acid** **(KYNA)**	- Can scavenge ROS in an N-methyl-D-aspartate receptor- and nicotinic receptor-independent way - In the concentration above 100 µM, it can abolish ROS production induced by iron(II) sulfate - Its excess can have detrimental effects on the function of the CNS cells		[12,13,96,97,98]
	This compound acts mostly as a neuroprotectant and exerts a wide spectrum of endogenous antagonism on ionotropic excitatory amino acid receptors and antioxidative properties. However, its excess can have detrimental effects on CNS functioning.		
**3-Hydroxykynurenine (3-HKYN)**	- Interacts with cellular xanthine oxidase, can generate superoxide radicals, hydrogen peroxide, and hydroxyl radical in amounts capable of inducing internucleosomal DNA damage - Participates in latter phases of cellular ROS production in response to inflammation; the early ROS production is not related to the mitochondrial alteration induced by 3-HKYN - Can decrease lipid peroxidation and reduced GSH oxidation in brain cortex homogenates - Promotes NAD^+^ synthesis at concentrations below 100 nM - In concentrations above 100 nM, it causes a decrease in intracellular NAD+ level and an increase in extracellular lactate dehydrogenase (LDH) activity - Exerts toxic effects through mechanisms that include impairment of cellular energy metabolism, which are not related to early ROS production - Acts with pro-oxidant actions at low (5–20 nM) concentrations, causes cell death in primary neuronal cultures prepared from rat striatum by the generation of hydrogen peroxide and hydroxyl radical, and can work as an antioxidant in higher (100 nM) concentrations and scavenge hydroxyl radicals and peroxynitrite	- Stimulates synthesis and activation of the Nrf2, the transcription factor, causing a partial increase in cell resistance against oxidative stress - Decreases mitochondrial membrane potential both in vitro in rat cultured cortical astrocytes and in vivo after intrastriatal injection into Wistar rats - Induces glutathione GST and SOD activities in rat brains	[58,99,100,101,102,103,104,105,106]
	Demonstrated both pro-oxidative and antioxidative effects. Physiologically, it is more likely to modulate redox activity and reduce the risk of cellular oxidative damage. This suggests that in physiological conditions 3-HKYN appears to be rather a redox modulator than a neurotoxin. However, alterations in its level may contribute to oxidative damage of cells.		
**3-hydroxyanthranilic acid (3-HAA)**	- Can scavenge hydroxyl radical and peroxynitrite - Can form complexes with iron(II), inhibiting its autoxidation, reducing ROS formation - Decreases mitochondrial membrane potential in vitro in rat cortical astrocyte cultures - Exerts toxic effects through mechanisms that include impairment of cellular energy metabolism, which are not related to early ROS production - Can decrease lipid peroxidation and reduced GS oxidation in brain cortex homogenates - Is prone to self-oxidation - Inhibits the mitochondrial respiratory chain reactions	- Decreases mitochondrial membrane potential in vivo after intrastriatal injection into Wistar rats - Induces glutathione GST and SOD activities, generating superoxide radicals, H_2_O_2_, and cinnabarinic acid	[11,103,107,108,109]
	Similar to 3-HKYN, shows both pro-oxidative and antioxidative properties and physiologically acts rather as redox modulator and antioxidant. Additionally, alterations in its level may enhance oxidative damage of cells.		
**Anthranilic acid (AA)** **Xanthurenic acid (XA)**	- Can form complexes with iron inhibiting its autoxidation - Blocks citric acid cycle and the respiratory chain complexes I–III, interfering with mitochondrial function - Has an anti-inflammatory effect by forming a complex with copper and acting as a hydroxyl radical inactivating ligand		[109,110,111,112]
	Revealed potent antioxidant and neuroprotective properties		
	- Demonstrated both pro- and antioxidative properties		[113,114]
	Demonstrated similar properties to its precursor, 3-HKYN, based on similar mechanisms		
**Picolinic acid (PIC)**	- has non-selective metal ion chelating and neuroprotective abilities		[115]
	Showed antioxidant and neuroprotective effects. Its efficacy against was lower than KYNA but higher than AA.		
**Quinolinic acid (QA)**	- Enhances Ca^2+^-induced ROS formation - Increases lipid and protein peroxidation - Forms complexes with iron, which induce the formation of hydroxyl radicals - Enhances ROS synthesis, inducing nitric oxide synthases and intracellular poly(ADP-ribose) polymerase activity and extracellular LDH activation	- Stimulates synthesis and activation of the Nrf2, the transcription factor, and causes a partial increase in cell resistance against oxidative stress - Can modify the synthesis and activity of certain endogenous antioxidants in the brain, such as reduced GS and SOD - Increases the severity of the lipid and protein peroxidation induced by 3-nitropropionic acid, and probably other pro-oxidants, dependent on the cellular Ca^2+^ level - Can form complexes with iron(II), which enhance the formation of hydroxyl radicals - Can generate toxic peroxide radicals and peroxynitrite, both after intrastratial and intrahippocampal injection	[11,93,116,117,118,119,120,121,122,123,124,125,126,127,128,129,130,131]
	Demonstrated both potent pro-oxidative and excitotoxic properties. Its accumulation is associated with deleterious effects within brain cells and plays an important role in the development of neurodegenerative disorders		
**Nicotinamide adenine dinucleotide (NAD)**	- Works as an electron carrier and cofactor in some redox reactions - Protects cells against energy depletion		[61,62,63,64]
	Is essential to maintain the energetic balance and proper cell viability and functions, especially in increased oxidative stress level conditions		

**Table 2:** Impact of enzymes of the kynurenine pathway on oxidative stress during the development of neurodegenerative processes.

Enzyme	Role		References
	In Vitro	In Vivo	
**Tryptophan 2,3-dioxygenase** **(TDO, EC 1.13.11.11)**	- Is necessary to maintain proper NAD^+^ intracellular level	- Can increase inflammation-associated oxidative stress level, leading to increase in depression, anxiety-related behavior, AD, and HD symptoms severity	[11,68,151,163,199,200]
**Indoleamine 2,3-dioxygenase (IDO, EC 1.13.11.17**	- Can utilize superoxide anion radical as both a substrate and a co-factor - Is necessary to maintain proper NAD^+^ intracellular level - Its activation increases cell viability during inflammation and decreases in astrocytes death ratio after exposure to hydrogen peroxide		[11,160,192,194,195,196,197,198]
**Kynurenine 3-monooxygenase (KMO, EC 1.14.13.9)**	- In inflammatory conditions its amount increases in mitochondrial mass - Its moderate activation increases NAD^+^ synthesis and promotes favorable bioenergetics processes - Its prolonged induction decreases the mitochondrial spare-respiratory capacity and increases ROS production - Its chronic overactivity leads to energy stores depletion and induces cell damage - Its functional interactions with PINK1, PRKN, and DRP1 implicate KMO in mechanisms associated with mitochondrial dysfunction in neurodegeneration, such as defects in mitochondrial morphology and mitophagy	- In a Drosophila S2R+ cell genome-wide RNAi screen, the KMO homologue cinnabar was identified as a modulator of mitochondrial morphology and the recruitment of the familial Parkinsonism-related protein Parkin to depolarized mitochondria	[160,163,201,202,203]
**Kynurenine aminotransferases (KAT, EC 2.6.1.7)**		- Its inhibition, although it decreases brain level of antioxidative KYNA, may enhance cognitive abilities	[73,204,205,206,207]
**3-hydroxyanthranilic acid 3,4-dioxygenase (3-HAO, EC 1.13.1.5)**		- Its inhibition leads to the decrease in QA synthesis and alleviates functional deficits in the experimental model of spinal cord injury	[208]

## Data Availability

Not applicable.

## Abbreviations

3-Hydroxyanthranilic acid	3-HAA
3-Hydroxyanthranilic acid 3,4-dioxygenase	3-HAO
3-Hydroxykynurenine	3-HKYN
α-Amino-3-hydroxy-5-methyl-4-isoxazolepropionic acid receptor	AMPAR
Adenosine diphosphonate	ADP
Adenosine triphosphate	ATP
Alzheimer’s disease	AD
Aminocarboxymuconatesemialdehyde	ACMS
Anthranilic acid	AA
Aryl hydrocarbon receptor	AhR
Blood–brain barrier	BBB
Calcium	Ca^2+^
Central nervous system	CNS
Iron(II)G protein-coupled receptor 35	Fe^2+^GPR35
Glutathione	GS
Glutathione S-transferase	GST
Huntington’s disease	HD
Indoleamine 2,3-dioxygenase	IDO
Kynurenic acid	KYNA
Kynureninase	KYNU
Kynurenine	KYN
Kynurenine 3-monooxygenase	KMO
Kynurenine aminotransferase	KAT
Kynurenine pathway	KP
Lactate dehydrogenase	LDH
Large neutral amino acid transporter 1	LAT-1
Nicotinamide adenine dinucleotide	NAD
N-methyl-D-aspartate	NMDA
Parkinson’s disease	PD
Picolinic acid	PA
Quinolinate phosphoribosylotransferase	QPRT
Quinolinic acid	QA
Reactive oxygen species	ROS
Superoxide dismutase	SOD
Tryptophan	TRP
Tryptophan 2,3-dioxygenase	TDO
Xanthurenic acid	XA
